# Study protocol of the cost-effectiveness comparison of two preventive methods in the incidence of caries: A randomized, controlled clinical trial

**DOI:** 10.1097/MD.0000000000016634

**Published:** 2019-07-26

**Authors:** Miguel Ángel Fernández-Barrera, Edith Lara-Carrillo, Rogelio José Scougall-Vilchis, América Patricia Pontigo-Loyola, Leticia Ávila-Burgos, Juan Fernando Casanova-Rosado, Alejandro José Casanova-Rosado, Mirna Minaya-Sánchez, Carlo Eduardo Medina-Solís

**Affiliations:** a Program of Doctorate in Health Sciences, Autonomous University State of Mexico, Toluca; b Academic Area of Dentistry of Health Sciences Institute, Autonomous University of Hidalgo State, Pachuca; c Advanced Studies and Research Center in Dentistry “Dr. Keisaburo Miyata” of Faculty of Dentistry, Autonomous University State of Mexico, Toluca; d Health Systems Research Centre, National Institute of Public Health, Cuernavaca; e Faculty of Dentistry, Autonomous University of Campeche, Campeche, México.

**Keywords:** dental caries, Mexico, oral health, preventive dentistry, schoolchildren, sealants

## Abstract

**Introduction:**

Dental caries is the most frequent oral disease worldwide and the main cause of tooth loss in children and young adults. One of the most frequently affected areas is the occlusal surfaces of the first permanent molars (FPM) due to their morphological complexity. At present, several preventive treatments can reduce the incidence of this disease in school populations. In Mexico, the most commonly used technologies are those derived from some presentation of fluoride; on the other hand, research on this topic has been limited.

**Objective:**

To determine the cost-effectiveness of two different methods for preventing the incidence of caries on the FPM of schoolchildren (6–8 years of age) from public primary schools.

**Material and methods:**

This is a randomized experimental design. Participants will be assigned to two treatment groups of 114 each. In the first group, pit and fissure sealants will be applied, whereas the second group will receive a fluoride varnish. The work will be carried out in schoolchildren that attend public elementary schools in the state of Hidalgo, Mexico. The result variable will be the incidence of caries and the total cost of each of the interventions will be calculated in order to calculate the intervention's cost-effectiveness.

**Conclusion:**

This work will allow us to compare the cost-effectiveness of the pit and fissure sealants and the fluoride varnish in order to determine which offers the best results.

## 1 Introduction

Dental caries is one of the most frequently encountered oral health problems worldwide. Official figures report that between 60% and 90% of children worldwide suffer from dental caries and that caries occur mainly among the most socioeconomically disadvantaged populations.^[1]^ It affects different spheres of people's lives and has a negative impact on people's quality of life, which is why it is considered a public health problem.^[2]^ Dental caries is a demineralization process of the hard tissues of the tooth, which generates a softening of the surface and subsurface of the enamel that, if it is not stopped, can generate a cavity and even cause the loss of the dental organ.^[3]^ One of the areas most affected by dental caries are the pits and fissures of the occlusal surfaces^[4,5]^; according to some researchers, these pits and fissures account for about 90% of all caries cases due to the presence of deep and narrow pits and fissures.^[6,7]^ Several studies indicate that occlusal surfaces are more susceptible to decay than smooth surfaces due to a lack of post-eruptive maturation and reduced mineral content.^[6–8]^

Concepts on the management of carious lesions have changed during the last few decades.^[9–11]^ Increasing knowledge about the importance of preventive dentistry and the management of incipient lesions has modified the dentist's typical protocols, specifically by promoting the current focus on less invasive treatments and trying to preserve the greatest amount of enamel for as long as possible.^[9–11]^ Topical fluorides, in their different forms (dentifrices, mouth rinses, gels, and varnishes), allow a local protective effect to be generated on the tooth surface by increasing the exposure time between the fluoride and tooth enamel.^[12,13]^ This increased exposure helps facilitate the remineralization process, which decreases the appearance of new carious lesions in the areas where it is applied. The use of fluorides contributes significantly to the prevention and management of carious lesions.^[12]^ Also, the evolution of dental materials enabled the improvement of preventive methods in dentistry, e.g., the changes in and new types of resins and their application, from the technique of acid etching introduced by Buonocore in 1955 and the development of acrylic resins by Bowen in 1953 to the materials that we know today.

The pit and fissure sealer (PFS) is a low-viscosity resin that penetrates deep pits and fissures. This preventive method reduces the risk that the pits and fissures of the occlusal surfaces become carious by generating a physical barrier that prevents the accumulation of biofilms within these areas.^[14]^ The PFS has been modified over time, resulting in improved retention to the dental surface and decreased working time, which facilitated PFS's application along with the change in coloration offered by some brands. Additionally, some of these products even have technology that allows them to release fluoride, thereby improving the resin's cariostatic actions.^[15]^

In Mexico, dental caries is a public health problem in schoolchildren^[16–25]^; however, the use of preventive methods other than fluoride in school populations has not been studied in terms of the costs involved in its use nor its results in terms of caries reduction.^[26]^ Almost no clinical trials have been published in this country on this topic, which opens up new research avenues that will, eventually, allow health planners to make decisions based on evidence. The objective of this work will be to determine the cost-effectiveness of two different preventive methods in decreasing the incidence of dental caries on the first permanent molars (FPM) of schoolchildren (from 6 to 8 years of age) from public primary schools.

## 2 Methodology

### 2.1 Study design

This is a randomized experimental design that will be carried out on the FPMs of schoolchildren from public elementary schools in the state of Hidalgo, Mexico. Start date: 2019–03-05.

Hidalgo is one of the 31 states, which, with Mexico City, comprise the 32 Federal Entities of Mexico. It is divided in 84 municipalities and its capital city is *Pachuca de Soto*. Hidalgo is one of the Mexican states that participated in the fluoridated domestic salt program implemented nationally in 1991. Mexico has no water fluoridation program at the national level; however, some states, including parts of Hidalgo, have water with naturally high levels of fluoride. This is not the case in Pachuca, the capital city of Hidalgo.^[27,28]^

### 2.2 Participants

The treatments will be carried out within selected public primary schools in selected municipalities of the state of Hidalgo with little access to oral health programs. Participants will be children between 6 and 8 years of age with at least one permanent molar that is healthy and fully erupted with high or moderate risk of caries according to the Scottish Intercollegiate Guidelines Network (SIGN).^[29]^

### 2.3 Selection criteria

To be considered to participate in the trial, each potential participant must meet the established inclusion and exclusion criteria. Some of the criteria require oral examination within the baseline measurement, which, may result in some children not participating in the intervention and, therefore, the study.

### 2.4 Inclusion criteria

Children must be from 6 to 8 years of age, belong to the selected public elementary schools, and:

- Have at least one first molar that is healthy and fully erupted;
- Whose parents sign the informed consent form; and
- Decide to participate in the intervention.

### 2.5 Elimination criteria

- Fixed orthodontic appliances;
- Disability;
- Systemic disease; or
- Any alteration of the oral cavity.

### 2.6 Trial intervention

The PFSs to be used in this study have been used for years in other populations. Children will be randomly assigned to either the PFS or varnish group and each subject will remain in their assigned group throughout the study.

#### 2.6.1 Pit and fissure sealant

The PFS that will be used for this study is Clinpro3M. This PFS changes color when being light-cured and has a low viscosity, which allows it to have excellent penetration inside the pits and fissures. It has fluoride release capability and is applied through the total enamel etching system.

The application of the sealant will be carried out within the public primary schools with the help of a portable tripod and using natural light. Sealers will be reviewed every 3 months for a period of 24 months. In cases of partial or total release, the sealant will be reapplied.

#### 2.6.2 Fluoride varnish

The varnish used in this study will be DuraphatColgate, which consists of a 5% fluoride varnish containing 22,600 ppm of fluoride suspended in a resin base. It is an easy product to apply since it adheres quickly in the presence of saliva and its color allows good visual control.

Once the baseline measurement is obtained, the application will be made within the selected schools with prior prophylaxis and during the first hour of classes to ensure that the children do not consume food until at least 2 h after the treatment, considering the time of the meal recess.

The revisions and reapplications will occur every 3 months during the follow-up period of 24 months.

### 2.7 Risks and adverse effects

The procedures used in this intervention are currently used as standard treatments for clinical care. The risks for the participants are minimal and basically the same as those of patients that are exposed to these procedures during routine clinical care. Although there are very few reported cases of allergies or hypersensitivity to these products, it will be necessary to question the parents about possible reactions to some of the compounds of the Duraphat varnish or the Clinpro sealer.^[29–32]^ Adverse events and other unintended effects of trial interventions will be reported to the committee of the university where the protocol was approved. The study will be audited every 6 months, and will be carried out by the IRB.

### 2.8 Measuring the effectiveness of the intervention

The primary results for this project are the incidence of caries at 6, 12, 18, and 24 months between the sealant and varnish groups.

The incidence of dental caries considers the measurement of the existence (or not) of visible changes in the surface of the enamel compared to the basal measurement. In the clinical evaluation, visual caries detection and a standardized and international caries measurement system, known as International Caries Detection and Measurement System (ICDAS), will be used.

The reviews will occur every 6 months for a 24-month period.

#### 2.8.1 International caries detection and measurement system (ICDAS)

ICDAS is a caries measurement system that uses the visual method to detect caries. It can measure carious lesions at the subclinical stage; ICDAS considers coloration changes sufficient to identify lesions in the incipient stages and does not recommend the use of explorers.

This measurement system examines five surfaces and uses codes that range from 0 to 6, depending on the severity of the injury, where 0 indicates a healthy surface.^[33–35]^ This system also evaluates the condition of the surface in terms of restorations, where it uses codes that range from 0 to 8, depending on the type of restoration used.^[33–35]^ For this study, the examiners will be calibrated and previously standardized (Kappa > 0.80).

### 2.9 Estimation of costs

The estimation of the costs of the interventions will be from the perspective of the service provider, i.e., all the human and material inputs necessary for the implementation of these two interventions will be identified and included. The unit and average cost of each one of the interventions will be estimated in the same evaluation periods; afterward, it will be determined which of the two interventions is more cost-effective for reducing the incidence of caries.

The cost estimate will include costs related to the training of the personnel that will apply each of the technologies, number of people needed for the application, salaries, and application time, number of control reviews, as well as the costs of purchasing the material and instruments.

### 2.10 Sample size

The sample size was calculated using a formula for the difference of proportions. The proportions used in this calculation were the decreases in incidence reported with the use of fluoride varnish (60%) and PFS (40%), a power of 80%, and an α of 95%, which gave a sample size of 97 subjects. In the end, 15% more subjects were added due to the losses to follow-up reported in longitudinal studies, which amounted to 114 subjects for each group.

### 2.11 Recruitment

The first thing that will be done in this process will be to obtain permission to conduct the study from the corresponding school authorities. For this, it is necessary to deliver a printed protocol to the school addresses of the area; once the study is authorized by the authorities, the following step will be to obtain permission from each of the schools that will participate in the intervention.

The next step is to obtain, through meetings with the parents, authorization for their children to participate in the investigation through the signing of informed consent forms.

Once informed consent has been obtained, the children will be reviewed to identify possible candidates for the study according to the inclusion and exclusion criteria. Once the established sample size has been reached, the participants will be randomly assigned to one of the intervention groups.

### 2.12 Assignment for each intervention group

For the randomization process, the total number of candidates will be included in an Excel database; Excel includes a random number function, which will be used in the randomization process.

### 2.13 Blinding and masking

In this study, the type of blinding that will be applied will be simple since the participants will not know what type of technology will be used in each individual. To guarantee this, the subjects will not observe the applications that the other participants will receive. This implies that the subjects will pass individually and be isolated from the other subjects during their participation. In addition, a very similar protocol will be carried out for the application, such as prophylaxis, and therefore the participant will not know what type of treatment will be applied.

### 2.14 Data collection and analysis

The information will be collected through primary sources of information, that is, structured odontograms for research. The data will be stored in an Excel database, where the cells will be censored to reduce errors during data capture. For the analysis, the database will be exported to Stata 14.0, where a univariate analysis will be carried out first and followed by a bivariate analysis to find associations between the dependent and independent variables. Non-parametric statistical tests will be used due to the variables’ measurement scale.

#### 2.14.1 Collection and analysis of the data regarding caries incidence

The incidence of dental caries will be analyzed using the ICDAS at 6, 12, 18, and 24 months after the intervention. The data will be collected by a researcher trained in this measurement system.^[34,35]^ The collected data will be captured according to the codes established in the ICDAS manual.

Caries status

0 = Sound

1 = First visual change in enamel (whitespot seen after 5 s air drying).

2 = Distinct visual change in enamel (whitespot seen without air drying).

3 = Localized enamel breakdown due to caries with no visible dentin

4 = Non-cavitated surface with underlying dark shadow from dentin

5 = Distinct cavity with visible dentin

6 = Extensive distinct cavity with visible dentin. An extensive cavity involves at least half of a tooth surface and possibly reaching the pulp.

7 = Tooth extracted because of caries (tooth surfaces will be coded 97)

8 = Tooth extracted for reasons other than caries (tooth surfaces will be coded 98)

9 = Unerupted (tooth surfaces coded 99)

In each of the measurements, the change occurred in the occlusal surfaces of the first permanent molars between the two preventive treatments used to determine which is more effective in preventing caries in each of the established periods.

#### 2.14.2 Analysis of the cost of the intervention

Once the direct cost of the interventions is obtained, the cost-effectiveness ratios will be generated as recommended in the literature.^[36]^

## 3 Discussion

Dental caries is one of the most frequent problems of the oral cavity. It affects more than half of the child population worldwide and is the leading cause of tooth loss in children and young adults.^[1]^ At present, there are different preventive methods that allow practitioners reduce the incidence of caries, among which the methods used in this intervention stand out; however, in Mexico, such work is still very scarce. The design of the present study will allow us to compare the effectiveness of both technologies and determine the total cost and cost-effectiveness of each. The information generated by this work can contribute to the decision-making of health planners.

In Mexico, the use of technologies that prevent oral diseases, other than those derived from fluoride, is limited, e.g., the low rate of use of PFSs reported by the Epidemiological Surveillance System for Oral Pathologies.^[26]^

The benefit of carrying out preventive programs in children is that they improve the subjects’ quality of life, reduce the risks of general disease, and reduce future out-of-pocket expenses for the population and health systems associated with the treatment of this disease.^[28,37]^ Results will be disseminated in Mexican and international scientific and professional venues.

## Author contributions

**Conceptualization:** Miguel Ángel Fernández-Barrera, Edith Lara-Carrillo, Rogelio José Scougall-Vilchis, América Patricia Pontigo-Loyola, Leticia Ávila-Burgos, Juan Fernando Casanova-Rosado, Alejandro José Casanova-Rosado, Mirna Minaya-Sánchez, Carlo Eduardo Medina-Solís.

**Data curation:** Miguel Ángel Fernández-Barrera, Carlo Eduardo Medina-Solís.

**Formal analysis:** Miguel Ángel Fernández-Barrera, Edith Lara-Carrillo, Rogelio José Scougall-Vilchis, Leticia Ávila-Burgos, Juan Fernando Casanova-Rosado, Alejandro José Casanova-Rosado, Mirna Minaya-Sánchez, Carlo Eduardo Medina-Solís.

**Funding acquisition:** América Patricia Pontigo-Loyola, Carlo Eduardo Medina-Solís.

**Investigation:** Miguel Ángel Fernández-Barrera, Edith Lara-Carrillo, Rogelio José Scougall-Vilchis, América Patricia Pontigo-Loyola, Leticia Ávila-Burgos, Juan Fernando Casanova-Rosado, Alejandro José Casanova-Rosado, Mirna Minaya-Sánchez, Carlo Eduardo Medina-Solís.

**Methodology:** Miguel Ángel Fernández-Barrera, Edith Lara-Carrillo, Rogelio José Scougall-Vilchis, América Patricia Pontigo-Loyola, Leticia Ávila-Burgos, Juan Fernando Casanova-Rosado, Alejandro José Casanova-Rosado, Mirna Minaya-Sánchez, Carlo Eduardo Medina-Solís.

**Project administration:** Miguel Ángel Fernández-Barrera.

**Resources:** Carlo Eduardo Medina-Solís.

**Supervision:** Miguel Ángel Fernández-Barrera, Edith Lara-Carrillo, Rogelio José Scougall-Vilchis, América Patricia Pontigo-Loyola, Carlo Eduardo Medina-Solís.

**Validation:** Miguel Ángel Fernández-Barrera, Carlo Eduardo Medina-Solís.

**Writing – original draft:** Miguel Ángel Fernández-Barrera, Edith Lara-Carrillo, Rogelio José Scougall-Vilchis, América Patricia Pontigo-Loyola, Leticia Ávila-Burgos, Juan Fernando Casanova-Rosado, Alejandro José Casanova-Rosado, Mirna Minaya-Sánchez, Carlo Eduardo Medina-Solís.

**Writing – review & editing:** Miguel Ángel Fernández-Barrera, Edith Lara-Carrillo, Rogelio José Scougall-Vilchis, América Patricia Pontigo-Loyola, Leticia Ávila-Burgos, Juan Fernando Casanova-Rosado, Alejandro José Casanova-Rosado, Mirna Minaya-Sánchez, Carlo Eduardo Medina-Solís.

Carlo Eduardo Medina-Solís orcid: 0000-0002-1410-9491.
